# Dataset from genome sequencing, assembly and mining of microsatellite markers in barred-button quail (*Turnix suscitator*)

**DOI:** 10.1016/j.dib.2023.109288

**Published:** 2023-06-01

**Authors:** Prateek Dey, Swapna Devi Ray, Padmanabhan Pramod, Ram Pratap Singh

**Affiliations:** a Sálim Ali Centre for Ornithology and Natural History, Anaikatty, Coimbatore, Tamil Nadu 641108, India; b Bharathiar University, Coimbatore, Tamil Nadu 641046, India; c Department of Life Science, Central University of South Bihar, Gaya 824236, India

**Keywords:** Whole-genome, SSR, Charadriiformes, Turnix

## Abstract

*Turnix suscitator* (barred-button quail) is a member of the primitive genus *Turnix* in the highly diverse order of shore birds Charadriiformes. Absence of genome scale data of *T. suscitator* has limited our understanding about its systematics, taxonomic and evolutionary history as well has hindered the characterization of genome wide microsatellite markers of the same. Hence we generated whole genome short read sequences of *T. suscitator*, created a high quality assembly and mined genome-wide microsatellite markers from the same. A total of 34142524 reads were sequenced with an estimated genome size of 817 mb. SPAdes assembly consisted of 320761 total contigs and an estimated N50 value of 907 base pairs. Krait identified a total of 77028 microsatellite motifs covering 0.64% of the total sequences in the SPAdes assembly. Further the whole genome sequence and genome wide microsatellites dataset of *T. suscitator* will facilitate future genomic/evolutionary studies of *Turnix* species.

**Specifications Table**

**Table:** 

Subject	Biological sciences and Zoology
Specific subject area	Wildlife genomics, avian genomics
Type of data	Sequence files (.gz) Table Image Chart Graph Fig.
How the data were acquired	High molecular weight genomic DNA of *Turnix suscitator* was extracted and used to generate pair-end genomic libraries using TruSeq DNA PCR-Free library preparation kit. Datasets for whole genome sequences of *Turnix suscitator* was sequenced using Illumina NextSeq550. Raw reads were assembled into contigs using SOAPdenovo2 and SPAdes. Microsatellite motifs from the assembly was mined using Krait.
Data format	Raw Analyzed Filtered
Description of data collection	Tissue sampled from *Turnix suscitator* road-killed specimen was used for DNA extraction, subsequent library preparation and whole genome sequencing. The raw paired-end reads were de-multiplexed, trimmed, quality controlled and assembled into contigs using suitable aligners. Whole genome assemblies were evaluated on various parameters and best assembly used to mine genome-wide microsatellite markers from the same.
Data source location	• Institution: Sálim Ali Centre for Ornithology and Natural History • City/Town/Region: Coimbatore, Anaikatti • Country: India
Data accessibility	Repository name: Mendeley Data and National Center for Biotechnology Information (NCBI) For Mendeley data Whole genome contig assemblies from SOAPdenovo2 and SPAdes aligners are stored in folder “Soapdenovo2_assembly” and “Spades_assembly” respectively. Output file from Krait software, containing information of the mined genome wide microsatellite motifs and its various attributes are stored in folder “Krait_output”, file name “Msat_BBQ_23092021.html”. Information regarding 50 most polymorphic microsatellite and its in-silico PCR amplification results are stored in folder “Krait_output”, file names “50_most_putative_primers” and “In_silico_validation” respectively. Microsatellite motif information for each primer is also stored in folder “Krait_output” under file name “In_silico_validation_with_motif_info”. Identifier DOI:10.17632/hfntj4vhpb.2 Direct link to folder: https://data.mendeley.com/datasets/hfntj4vhpb/2 National Center for Biotechnology Information (NCBI) Datasets and information on the whole genome paired-end sequences can be obtained from: BioProject, BioSample and SRA numbers are PRJNA807611, SAMN25996056 and SRR18050964 Direct link to data: https://www.ncbi.nlm.nih.gov/bioproject/PRJNA807611
Related research article	NA

## Value of the Data

• Information regarding the life history, ecological and genomic attributes of wild *T. suscitator* species has been seldom revealed and information is rather scanty. In this aspect, no attempts have been made either to generate genomic data or identify genome-wide microsatellite markers of *T. suscitator* species. Absence of genome scale data and microsatellite markers of *T. suscitator* has created huge gaps in deciphering systematics/taxonomic and evolutionary queries of such an enigmatic species.
• Researchers working in the field of wildlife genomics and conservation genetics will benefit greatly from the data.
• The genome survey data and microsatellites identified in this study will be of practical assistance in development of useful molecular markers and high-level genome assembly of a number of *Turnix* species in the future. Overall, the information generated in this study will not only be instrumental in designing evolutionary/phylogenetic/phylogeography/population studies in *T. suscitator* but also in congeneric species of Turnicidae family.

## 1 Objective

*Turnix suscitator* (Barred-button quail) of family Turnicidae are a group of small palaeotropical birds in the order Charadriiformes [1]. Although *T. suscitator* has a large geographic distribution, behaviourally the species is shy and elusive. Hence, studies on genetics and genomic attributes of wild *T. suscitator* has been rarely reported, and information regarding the same is scarce [1]. Further no genomic data or genome-wide microsatellite marker information of *T. suscitator* species exists. Absence of genome scale data of *T. suscitator* has limited our understanding about its systematics, taxonomic and evolutionary history as well has hindered the characterization of genome wide microsatellite markers of the same [2,3]. Advances in massively parallel next generation sequencing (NGS) technology has made sequencing of non-model organism like *T. suscitator* relatively less arduous [4]. Hence, this dataset was generated to reveal whole genome sequences of *T. suscitator* species for the first time. The objective of generating this dataset is to create high quality assembly from the sequences and mine species specific microsatellite markers from the same.

## 2 Data Description

**Fig. 1:**
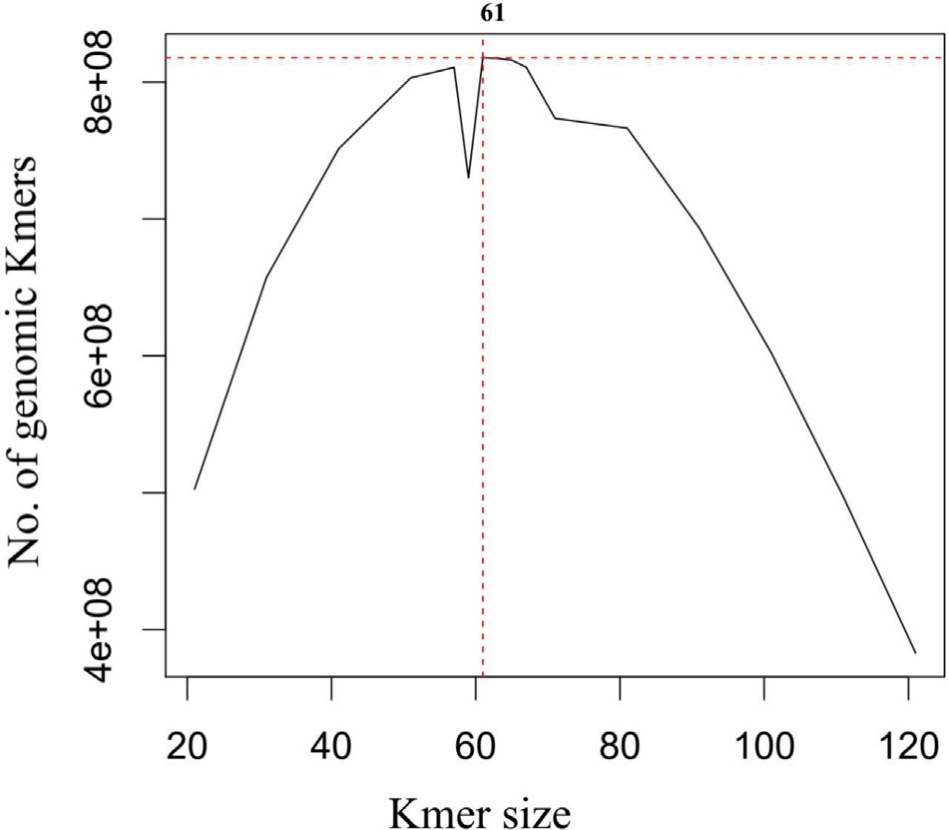
Kmer analysis for estimating best Kmer peak and genome size of *T. suscitator*. The best Kmer peak was estimated at 61

After successful sequencing in NextSeq550 we retrieved ∼12.5 giga bytes of paired end reads (350 base pair inserts). The 12.5 giga bytes comprised of 34142524 sequences (2 * 17071262 paired end reads). GC percentage of the raw reads was calculated at 63.5% and average read length calculated at ∼100 - 151 base pairs. All the sequences passed quality control test of FastQC and none of the sequences were flagged as poor quality. KmerGenie estimated the best Kmer size at 61 (Fig. 1). The genome size was estimated to be of 817821305 base pairs (bp) or 817.82 mega base pairs (mb). The reported raw paired-end reads has been deposited at National Center for Biotechnology Information (NCBI) GenBank under the BioProject accession number PRJNA807611.

Seqtk successfully filtered out the raw reads with Q score 30 for further analysis. The *denovo* genome assembly constructed through SOAPdenovo2 (at a Kmer size of 61) is deposited at Mendeley repository within folder Soapdenovo2_assembly under the file name BBQ_SD2_61kmer_contig.fasta. The SPAdes *denovo* assembly (employing multi Kmer approach) is stored at Mendeley repository inside folder Spades_assembly under the file name BBQ_Spades_contig.fasta. The assemblies henceforth are mentioned as SOAPdenovo2 and SPAdes assembly respectively. QUAST-LG analysis revealed SOAPdenovo2 assembly consisted of 190400 total contigs, with largest contig being 4183 base pair in length, GC% of 47.64 and N50 value of 453 base pairs (Table 1). Similarly QUAST-LG analysis of SPAdes assembly consisted of 320761 total contigs with largest contig being 16839 base pair in length, GC% of 46.13 and N50 value of 907 base pairs (Table 1). Almost all the parameters evaluated through QUAST-LG estimated SPAdes assembly to be the best amongst the two assemblies and the same was utilized for microsatellite mining and identification.

**Table 1:** The assemblies of *T. suscitator* sequences created through SOAPdenovo2 and SPAdes softwares were compared and evaluated for various parameters using QUAST-LG. This table details the comparative assembly statistics of the same.

	SOAPdenovo2	SPAdes
No. of contigs	190400	320761
Largest contig	4183	16839
Total length (bp)	87481216	238075843
GC (%)	47.64	46.13
N50	453	907
N75	362	502
Number of N's per 100 kilo base pair	0.00	0.04

**Fig. 2:**
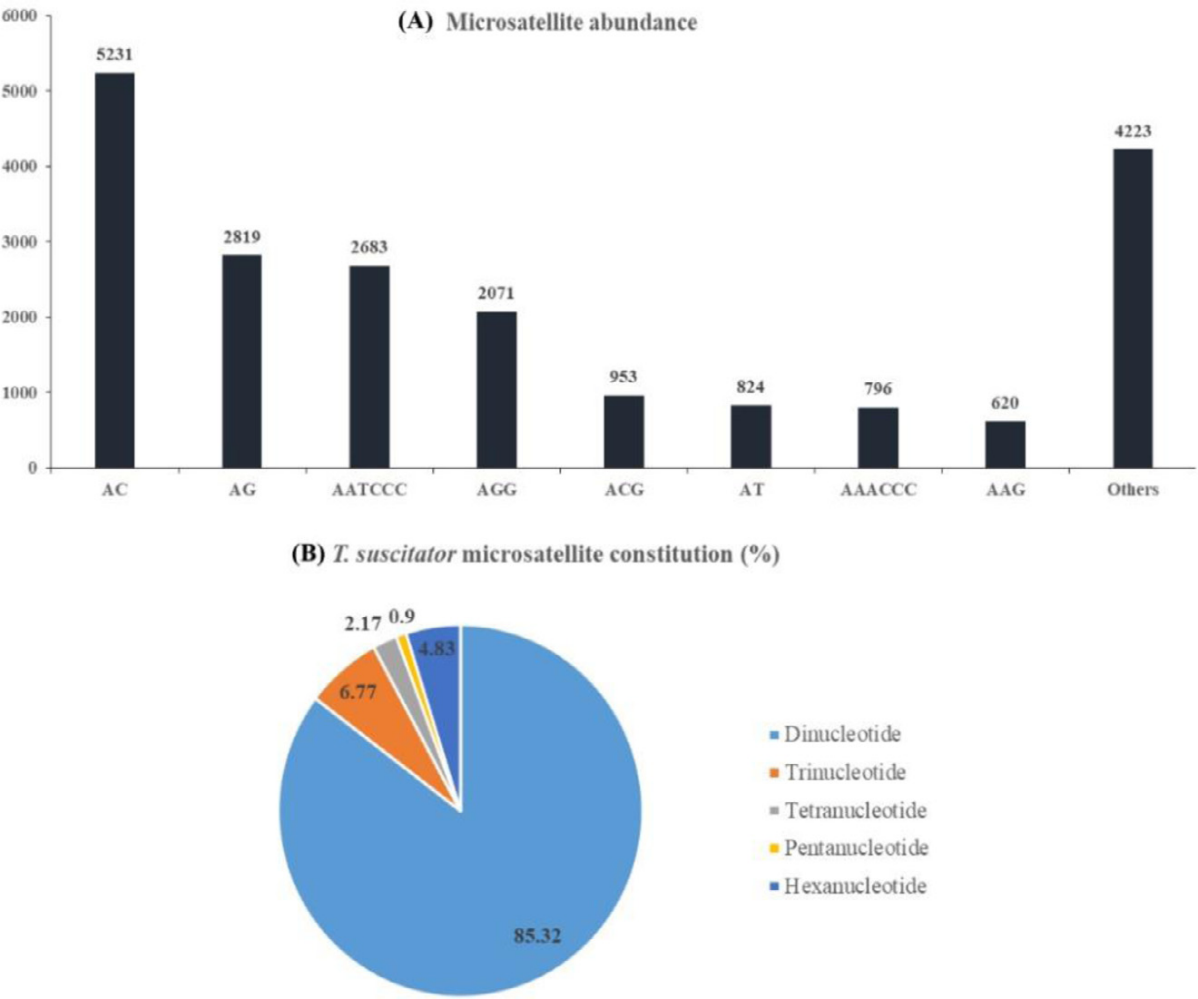
Characterization of genome wide microsatellites of *T. suscitator*. (A) Most abundant motif repeat types in the identified microsatellites. (B) Distribution of different types of microsatellites

Mining of microsatellite markers from raw reads may yield low number/quality of microsatellite motifs. Hence, we utilized high quality genome assemblies to mine for microsatellite markers of *T. suscitator* in this study. Krait identified a total of 77028 microsatellite motifs in the SPAdes genome assembly (Table 2). The output from Krait software is stored at Mendeley under the folder Krait_output with file name as Msat_BBQ_23092021.html. From the output, the average length of microsatellite motif was estimated at 25.64 base pair and covered 0.64% of the total sequences in the assembly. Relative abundance (total microsatellite motifs/total sequence length of assembly) was calculated at 258.26 microsatellite per mb of the genome assembly and relative density (total length of microsatellite motifs/total sequence length of assembly) was estimated at 6621.2 base pair per mb (Table 2). Of the mined microsatellite motifs, dinucleotide constitute the highest 65722 (85.32%), trinucleotide 5218 (6.77%), tetranucleotide 1674 (2.17%), pentanucleotide 693 (0.9%) and hexanucleotide 3719 (4.83%) numbers respectively (Fig. 2). The most abundant repeats amongst the mined motifs are detailed as AC, AG, AATCCC, AGG, ACG, AT, AAACCC, AAG respectively (Fig. 2). Primers were designed from sequences adjoining the identified microsatellites. A set of 50 most putative microsatellites (selected following the criteria mentioned in methods section) were validated in-silico through FASTPCR and deposited at Mendeley under the folder Krait_output with file name as 50_most_putative_primers.txt. All the primer pairs showed verifiable amplification, with 16 primer pairs amplifying into multiple amplicons and 34 into single amplicon. Validation details stored at Mendeley under the folder Krait_output with file name as In_silico_validation.txt. All associated files such as whole genome assemblies, microsatellite mining and in-silico results are stored at Mendeley under under doi:10.17632/hfntj4vhpb.2.

**Table 2:** *T. suscitator* SPAdes assembly was utilized to mine microsatellite motifs using Krait software. Various attributes of microsatellite motifs mined from the same are presented in this table.

Attribute	Value
Total number of perfect microsatellite motifs	77028
The average length of microsatellite motifs	25.64
The percentage of sequence covered by microsatellites	0.67
Relative abundance (total microsatellite motifs/total sequence length of assembly)	258.26 microsatellite per mega base pair of the genome assembly
Relative density (total length of microsatellite motifs/total sequence length of assembly)	6621.2 base pair per mega base pair of the genome assembly

## 3 Experimental Design, Materials and Methods

### 3.1 Study area and Sample collection

Road-killed sample of *T. suscitator* was obtained with due permission from Maharashtra Forest Department (Desk-22(8)/Research/CR-8(18-19)/875/2018-19). Tissue was sampled and stored in DESS buffer (20% w/v DMSO, 0.25 M w/v tetra-sodium EDTA, Sodium Chloride (∼40 g/100 ml ddH2O till saturation, pH 7.5). About 20 mg of the muscle tissue was digested using lysis buffer (10 mM w/v Tris-pH 8.0, 10 mM w/v EDTA-pH 8.0, and 100 mM w/v NaCl) in addition to 40 μl of 20% w/v SDS and 40 μl of Proteinase K (25 mg/ml) enzyme. DNA extraction from the digested lysate was performed using modified Chloroform and Isoamyl alcohol method [5]. The extracted DNA of the specimen was deposited in the Avian Biobank facility at NAFL under the voucher code (NAFL/0325/DNA/180920). The quality of the extraction was assessed using 1% agarose gel and quantified through spectrophotometer (DeNovix, USA) and Qubit 4 Fluorometer (ThermoFisher Scientific, USA) utilizing Qubit 1X ds DNA HS kit (Invitrogen, USA).

### 3.2 Library preparation

High molecular weight genomic DNA was used to generate pair-end genomic libraries of insert size 350 (2×150) base pairs using TruSeq DNA PCR-Free library preparation kit (Illumina Inc., USA). Library preparation was done following TruSeq DNA PCR-Free library preparation kit protocol. About 1100 nanogram of the isolated DNA was used as starting material to generate paired-end genomic library of insert size 350 (2×150) base pairs. Focused ultrasonicator (Covaris M220, USA) was used to fragment the genomic DNA to desired fragment size. Subsequently TruSeq DNA PCR-Free library preparation kit was used to clean-up the fragmented DNA, create blunt-ends and ligate adapters to the library fragments performed. Previous works outline the detail methodology for library preparation [6,7]. Upon successful library preparation mean peak size of the pair-end library created was assessed using Fragment Analyzer AATI 5200 (Agilent, USA) and quantified using QIAseq Library Quant Assay Kit (Qiagen N.V., Germany). The library was sequenced using NextSeq550 (Illumina Inc., USA) and at the end of the sequencing run, high-quality paired-end reads were obtained.

### 3.3 Processing of raw reads and De-novo genome assembly

The raw reads generated from NextSeq550 was de-multiplexed and trimmed of adapters using bcl2fastq v2.20.0 software (Illumina Inc., USA). Quality control of the raw sequencing reads was done through FastQC v0.11.8 (https://www.bioinformatics.babraham.ac.uk/projects/fastqc/). Further downstream analysis was performed only on reads passing Phred (Q) score threshold of 30 or above, using Seqtk v1.3(r106) software [8]. High quality pair-end reads with a Q score of 30 or above were utilized for de-novo genome assembly of *T. suscitator*. K-mer analysis on peak depth and best predicted K-mer selection from the high quality raw sequence reads was performed using KmerGenie v1.7051 [9]. At first, predicted K-mer size was used to de-novo assemble *T. suscitator* genome using SOAPdenovo2 v2.04 [10]. Secondly a multi K-mer approach was employed using SPAdes v3.14.0 to assemble the high quality pair-end reads into a separate genome assembly [11]. Both the assemblies were compared and evaluated for various parameters using QUAST-LG v5.0.2 [12].The assembly scoring highest on all parameters was used for further microsatellite mining.

### 3.4 Microsatellite mining, primer design and in-silico marker validation

The best assembly selected through the above mentioned criteria was used for microsatellite motif identification, i.e. SPAdes assembly. The reads of the *de-novo* SPAdes assembly was used as an input for Krait v1.3.3 [13]. Krait was employed to mine all potential microsatellite motifs using search parameters used for detection of di-, tri-, tetra-, penta-, and hexa- nucleotide motifs were set at a minimum of 6, 5, 5, 5, 5 repeats respectively and *Primer3* embedded in Krait were used for designing primers flanking the microsatellite region. A criteria was optimised to select 50 most polymorphic primers from the total microsatellites mined. The criteria was devised prioritising high repeat numbers, equal annealing temperature (59˚C-61˚C) and pure sequence repeats, such that 50 most probable polymorphic and stable microsatellites were chosen. In-silico validation of the select 50 microsatellites was carried out using FastPCR v6.7 software [14].

## Ethics Statements

Not applicable. Research did not include human or live animal subjects.

## CRediT authorship contribution statement

**Prateek Dey:** Methodology, Investigation, Writing – original draft, Data curation, Visualization, Software. **Swapna Devi Ray:** Investigation, Data curation, Software. **Padmanabhan Pramod:** Writing – review & editing, Supervision, Project administration, Funding acquisition. **Ram Pratap Singh:** Conceptualization, Writing – original draft, Writing – review & editing, Supervision, Project administration, Funding acquisition.

## Declaration of Competing Interests

The authors declare that they have no known competing financial interests or personal relationships that could have appeared to influence the work reported in this paper.

## Data Availability

BBQ_wgs_DatainBrief2023 (Original data) (Mendeley Data). Turnix suscitator_WGSS (Original data) (NCBI).
